# A biotin-streptavidin-biotin bridge dramatically enhances cell fusion

**DOI:** 10.3892/ol.2014.2067

**Published:** 2014-04-15

**Authors:** JINHUA LI, XIANZHONG YU, THOMAS E. WAGNER, YANZHANG WEI

**Affiliations:** 1 Department of Biotechnology, Greenville Technical College, Greenville, SC 29601, USA; 2 Department of Biological Sciences, Clemson University, Clemson, SC 29634, USA; 3 Orbis Health Solutions, LLC., Greenville, SC 29601, USA

**Keywords:** biotin, streptavidin, polyethylene-glycol, electroporation, cell fusion

## Abstract

Although the generation of hybrid cells by cell fusion plays a significant role in biotechnology and biomedicine, the low cell-fusion rates and the limitation of large-scale cell fusion for clinical applications of the two widely used approaches, polyethylene-glycol (PEG)-mediated cell fusion and electrofusion, hinder the application of this critical technology in certain key areas, including cancer immunotherapy. In the present study, a simple procedure that can not only significantly increase the heterologous cell fusion but is also capable of producing fused cells on a large scale is reported. A biotin-streptavidin-biotin (BSB) bridge was created by coating one to-be-fused cell with biotin and the other with biotin-streptavidin. The BSB bridge enhances cell-fusion rates induced with PEG fusion or electrofusion by 10–30% depending on the cell types when compared with cell fusions without the bridge. The procedure described increases heterologous cell pairing and eliminates the alignment step required for the majority of electrofusions. Notably, it can be used to make large-scale cell fusions for clinical applications.

## Introduction

The generation of hybrid cells by cell fusion plays a significant role in biotechnology and biomedicine. It has been used for various purposes (1–8), among which the most successful has been the production of hybridomas to generate monoclonal antibodies (9,10). Recently, the technique has found its novel application in fusing dendritic cells (DCs) with tumor cells for cancer immunotherapy (11–13). Cell fusion can be induced in three main ways; virus-mediated cell fusion (14), polyethylene glycol (PEG)-induced cell fusion (15,16) and electric-pulse-induced cell fusion or electrofusion (17). Although virus envelope-mediated cell fusion often generates a higher cell-fusion rate (18,19), its use in therapeutic applications is limited due to the viral proteins. PEG-mediated fusion is widely used due to the simplicity of its procedure. However, the method often generates less hybrid cells, even when chemical conjugates have been introduced (20,21). Electrofusion has also been widely used recently to fuse cells, and methods to increase the heterologous cell fusion have been proposed (22,23), however, its use in large-scale clinical applications is limited.

We hypothesized that a biotin-streptavidin (SA)-biotin (BSB) bridge built between two to-be-fused cells will physically pull the two cells together and dramatically increase heterologous cell fusions induced by PEG or electroporation. The idea was tested in various types of cells.

## Materials and methods

### Mice and cells

Female C57BL/6J mice at 6–8 weeks of age were purchased from the Jackson Laboratory (Bar Harbor, ME, USA). The mice were housed in a pathogen-free animal facility. The animal experiments were carried out in accordance with the Guidelines for the Care and Use of Laboratory Animals (NIH Publication number 85-23) and the institutional guidelines of Clemson University (Clemson, SC, USA). The study was approved by the ethics committee of Clemson University. Two mouse tumor cell lines, B16F0 melanoma cells [CRL-6322; American Type Culture Collection (ATCC), Manassas, VA, USA] and S180 sarcoma cells (TIB-66; ATCC) were used. The cells were cultured in Dulbecco’s modified Eagle’s medium (Life Technologies, Grand Island, NY, USA) supplemented with 10% fetal bovine serum (FBS; Life Technologies). All cell culture media contained 100 μg/ml gentamicin (Life Technologies) and cells were cultured at 37°C with 5% CO_2_. Bone marrow-derived DCs were cultured as previously described (24). Briefly, bone marrow cells were flushed from the femur and tibia bones of female C57BL/6J mice with RPMI 1640 and passed through a 40 μm cell strainer. Following the removal of the red blood cells by lysis, using ammonium-chloride-potassium lysing solution [0.15 M NH_4_Cl, 1 mM KHCO_3_ and 0.1 mM Na_2_EDTA (pH 7.3)] at room temperature for 5 min, the bone marrow cells were suspended in DC medium containing RPMI-1640 (Gibco-BRL, Carlsbad, CA, USA), 10% FBS, 50 mg/ml gentamicin and 20 ng/ml recombinant murine granulocyte-macrophage colony-stimulating factor (rmGM-CSF) (Sigma Aldrich, St. Louis, MO, USA) and seeded into 100 mm bacterial culture petri dishes at a concentration of 2×10^6^ cells/10 ml/100 mm dish. At day 3, 10 ml of fresh DC medium was added into each dish. At day 6, half of the medium was removed and replaced with fresh DC medium containing 10 ng/ml rmGM-CSF. At day 8, the cells were centrifuged at 500 × g for 5 min and resuspended in fresh DC medium with 10 ng/ml rmGM-CSF, 100 ng/ml murine tumor necrosis factor-α (Sigma Aldrich) and 1 mM prostaglandin E2 (Sigma Alrdich). At day 10, the non-adherent cells (>70% mature DCs) were collected and were ready for further studies.

### Biotin labeling and dye staining

Prior to labeling, 10 million tumor cells in a T75 flask or 10 million DC cells in a 100-mm petri dish were washed twice with PBS. The cells were then labeled with biotin by adding 2 μl of N-hydroxysuccinimide-dPEG24-biotin (25 mg/ml; Quanta Biodesign, Ltd., Powell, OH, USA) into 10 ml PBS and incubating at 4°C for 40 min. Subsequent to biotinylation, the cells were washed twice with PBS. The biotinylated tumor cells were stained red with PKH26 dye or green with PKH67 dye (Sigma Aldrich), and DCs were stained green with PKH67 dye, according to the manufacturer’s instructions. Following the dye labeling and washing, the DCs were resuspended in PBS. The dye labeled-B16F0 cells were irradiated at 100 Gy, washed once with PBS and resuspended in 5 ml of PBS. For certain experiments, the cells were only stained with the fluorescent dyes without biotinylation.

### SA connection

Specific biotinylated cells were further labeled with SA. In order to be certain that all the biotin molecules on the cell were occupied by SA, an excess amount of SA was used. A total of 1 mg purified SA was added to 10 million cells in 10 ml PBS and incubated at 4°C for 20 min with occasional gentle mixing by shaking. The cells were then washed twice with PBS to remove the unbounded SA and resuspended in PBS. In order to prevent cell pairing or clustering at this step, an excess amount of SA was added to relatively diluted cell suspensions (~10 ml PBS was used for 10 million cells).

### Cell fusion

Biotin or biotin-SA labeled, green or red dye-stained cells were mixed at a ratio of 1:1 in PBS and incubated for 30 min at room temperature for biotin-SA binding on the cells to occur. The cell fusion was induced by the standard PEG method or electroporation without the alignment step. For electroporation, 0.7 ml of cell mixture was aliquoted into 4 mm gap BTX cuvettes and subjected to electroporation (450V, 60 μs, twice with 200 ms intervals) using a BTX model ECM830 electroporator (BTX Harvard Apparatus, Holliston, MA, USA). The fused cells were collected and placed in T75 flasks with appropriate culture media for later use. Virus envelope-mediated cell fusions were performed using the HVJ Envelope Cell Fusion kit, GenomOne-CF EX, according to the manufacturer’s instructions (Cosmo Bio Co., Ltd., Tokyo, Japan). The dye labeling and cell-fusion rates were evaluated under a fluorescent microscope or by fluorescence-activated cell sorting (FACS) analysis.

### Statistical analysis

One way analysis of variance with Bonferroni post-test were performed using the built-in software provided with GraphPad Prism^®^ 4 (GraphPad, La Jolla, CA, USA).

## Results and Discussion

The induction of cell fusion is critically important in biomedical research and clinical practice. Its applications are, however, hindered by the limitation of large scale production and relatively low fusion rate. Although efforts have been made to improve the fusion rate by using chemical conjugates (20,21) or increasing the heterologous cell fusion (22,23), optimal conditions for cell fusion remain to be established. In this study, a BSB bridge enhanced cell fusion rates induced with PEG-mediated fusion or electrofusion by 10–30% depending on cell types when compared with cell fusion without the bridge. The procedure increased heterologous cell pairing and eliminated the alignment step required for current electrofusion. More importantly, it can be used to make large scale cell fusions for clinical applications.

### BSB bridge enhances cell fusion

**Figure 1:**
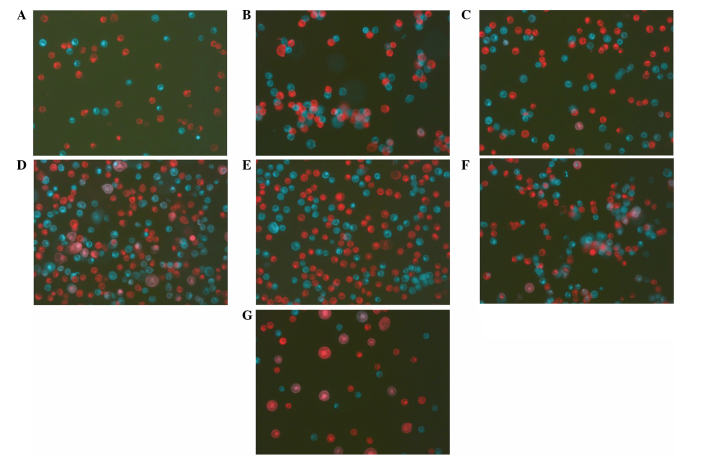
Biotin-SA-biotin bridge enhances cell fusions between tumor cells. (A) B16F0 cells were stained with green fluorescent dye, PKH67, or red fluorescent dye, PKH26, and mixed together. Cell fusions were then induced by (C) PEG, (E) electroporation or (G) viral envelope. By contrast, B16F0 tumor cells were first coated with biotin. Half of the cells were stained with PKH67 and the other half were stained with PKH26 and then treated with an excess amount of SA. (B) The green cells with biotin were mixed with red cells with biotin-SA and cell fusions were induced by (D) PEG or (F) electroporation. SA, streptavidin; PEG, polyethylene-glycol.

**Figure 2:**
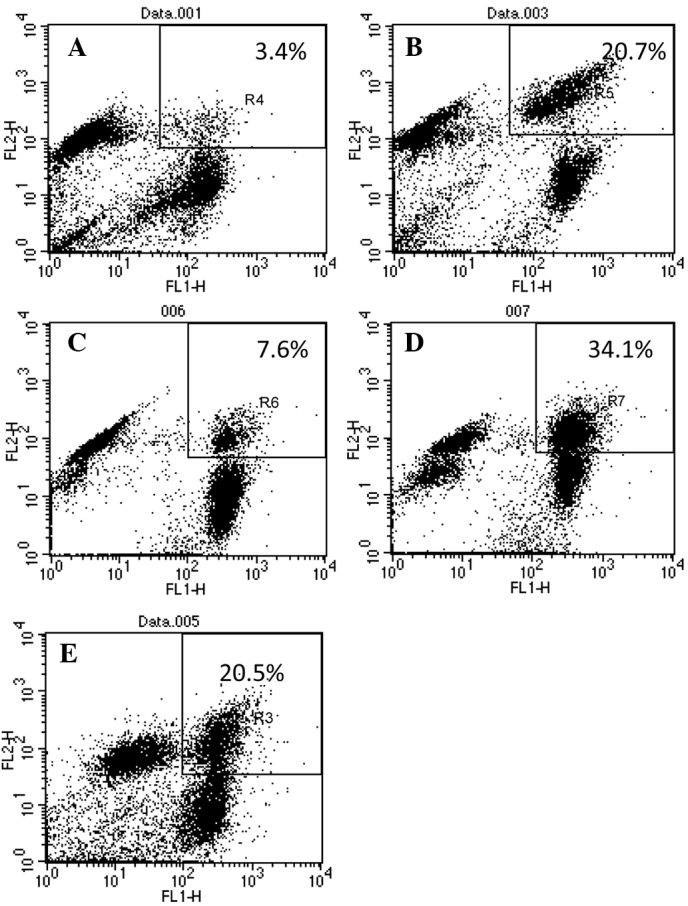
Fluorescence-activated cell sorting analysis of biotin-SA-biotin bridge-mediated cell fusion. B16F0 cells were either stained with green fluorescent dye, PKH67, or red fluorescent dye, PKH26, and mixed together. Cell fusions were then induced by (A) PEG, (C) electroporation or (E) viral envelope. By contrast, B16F0 tumor cells were first coated with biotin. Half of the cells were stained with PKH67 and the other half were stained with PKH26 and then treated with an excess amount of SA. The green cells with biotin were mixed with red cells with biotin-SA and cell fusions were induced by (B) PEG or (D) electroporation. SA, streptavidin; PEG, polyethylene-glycol.

To evaluate the idea of using the BSB bridge to enhance the cell-fusion rate, mouse melanoma B16F0 tumor cells were coated with biotin first and half of the cells were stained with a green fluorescent dye, PKH67, and the other half with a red fluorescent dye, PKH26. The red cells were then treated with SA in an excess amount to ensure that all the biotins on a cell were occupied. This step is significant as it first prevents the bridge formation between red cells and more importantly, there will be unoccupied biotin binding sites on SA available for binding biotins on the other cell type as each SA has four biotin binding sites. The green cells with biotin alone and the red cells with biotin-SA were mixed together at a ratio of 1:1. While there was no pairing in cell mixtures without a BSB bridge (Fig. 1A), within 5 min, the cells with a BSB bridge were paired (Fig. 1B) between green and red cells and there were no green-green or red-red pairings. The possibility of cell-cluster formation (more than two cells clustered together) can be decreased by adjusting the cell concentration in the mixture. Cell mixtures were then treated with the two most common cell-fusion methods; PEG and electroporation without alignment. Compared with the fusions without BSB bridges (Fig. 1C and 1E), the bridge significantly increased the fusion rate by PEG (Fig. 1D) or by electroporation (Fig. 1F). FACS analysis quantitatively confirmed the observation (Fig. 2) and showed that the fusion rate increased from 3.58±0.73 to 20.62±0.72% for PEG fusion, and from 5.98±0.57 to 31.44±1.69% for electroporation (Table I). It is significant to point out that the electroporation was performed using a simple electroporator (BTX model ECM830). Current electrofusions not only use more expensive machines, including BTX ECM 2001, but also require a cell-alignment step, which dramatically hinders its use in large-scale clinical applications. It is widely accepted that the Hemagglutinating virus envelope-mediated cell fusion generates the highest cell-fusion rate (14). As a control in the present study, green and red cells without the BSB bridge were fused using the HVJ Envelope Cell Fusion kit, GenomOne-CF EX and the fusion rate was 20.43±1.0% (Table I, Fig. 1G and 2E). Although, with the BSB bridge, PEG induced a similar fusion rate as the viral envelope (20.62±0.72 and 20.43±1.0%; Table I), and electroporation induced a significantly higher fusion rate (31.44±1.69%) compared with the viral envelope-induced fusion rate (20.43±1.0%). Therefore, the BSB bridge-mediated cell fusion is equivalent or superior to the virus envelope-mediated cell fusion.

### BSB bridge enhances DC/tumor cell fusion

**Figure 3:**
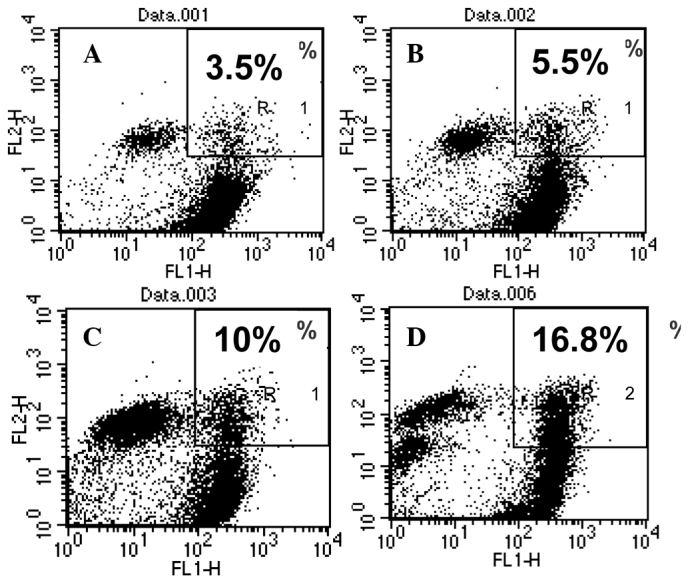
BSB bridge increases cell-fusion rate between DCs and tumor cells. DC/tumor fusions were induced by (A and C) PEG or (B and D) electroporation, (A and B) without or (C and D) with a BSB bridge. BSB, biotin-streptavidin-biotin; DC, dendritic cell; PEG, polyethylene-glycol.

To determine if the BSB bridge can increase the fusion rate of cells that are significant in biomedical applications, DCs were cultured from mouse bone marrow, coated with biotin and stained with green fluorescent dye PKH67, and mouse B16F0 tumor cells were coated with biotin, stained with red fluorescent dye PHK26 and treated with SA. The two cells were mixed at a ratio of 1:1 and fused using PEG or electroporation. In the control groups, DCs were stained green and tumor cells were stained red without the bridge. The results showed that the BSB bridge significantly increased the DC/tumor cell fusion rate from 2.88±0.56% to 12±1.42% for PEG fusion and from 4.08±0.5% to 16.64±0.46% for electroporation (Table I, Fig. 3). To evaluate the technology in other cells, lymphocytes were isolated from mouse spleen, coated with biotin and stained with red fluorescent dye PKH26. Following treatment with an excess amount of SA, the red lymphocytes were mixed with green B16F0 cells coated with biotin and fused with PEG or electroporation. The results (Table I) indicated that the BSB bridge significantly increased the fusion rate induced by PEG or electroporation. Notably, with the BSB bridge, electroporation induced more DC/B16F0 fusions compared with PEG (16.64±0.46% vs. 12±1.42%; p<0.001), while PEG induced more lymphocyte/B16F0 fusions compared with electroporation (36.78±2.82% vs. 21.38±1.70%; p<0.001) (Table I).

In conclusion, the coating of to-be-fused cells with biotin or biotin-SA and the formation of a BSB bridge significantly increases cell-fusion rates induced by PEG or electroporation in various types of therapeutically significant cells. Furthermore, the BSB bridge decreases self-self fusions and eliminates the cell-alignment step required for current electrofusion. Therefore, this simple improvement in technology will encourage more applications of cell fusion in therapeutic development.

## Figures and Tables

**Table I:** Summary of cell fusions.

	Fusion rate without BSB bridge (%)			Fusion rate with BSB bridge (%)	
		
Cells	PEG	Electroporation	Virus	PEG	Electroporation
B16F0/B16F0^a^	3.4	7.6	20.0	20.7	34.1
	4.6	5.4	18.9	19.6	30.0
	2.1	5.8	22.4	21.9	29.8
	5.8	6.8	-	18.5	36.4
	2.0	4.3	-	22.4	26.9
	3.58±0.73	5.98±0.57	20.43±1.0	20.62^b^±0.72	31.44^c,h^±1.69
DC/B16F0	3.5	5.5	22.0	10.0	16.8
	2.2	2.6	23.7	12.3	15.0
	4.1	3.3	19.4	9.8	17.5
	1.7	4.2	-	15.9	17.5
	0.0	4.8	-	0.0	16.4
	2.88±0.56	4.08±0.52	21.7±1.25	12^d^±1.42	16.64^e,i^±0.46
Lymphocyte/B16F0	1.6	1.9	18.9	43.3	22.3
	2.2	2.6	22.6	37.8	19.8
	3.1	2.5	17.8	29.6	25.7
	0.9	1.7	-	36.4	17.9
	1.95±0.46	2.18±0.22	19.77±1.45	36.78^f^±2.82	21.38^g,j^±1.70

^a^All the cells were mixed at a 1:1 ratio; ^b^BSB bridge significantly increased the fusion rate of tumor/tumor cells induced by PEG (p<0.001) or ^c^electroporation (p<0.001); ^d^BSB bridge significantly increased the fusion rate of DC/tumor cells induced by PEG (p<0.001) or ^e^electroporation (p<0.001); ^f^BSB bridge significantly increased the fusion rate of lymphocyte/tumor cells induced by PEG (p<0.001) or ^g^electroporation (p<0.001). With the BSB bridge, electroporation induced a higher fusion rate among tumor cells compared with PEG (^h^p<0.001), while the two methods did not show a difference in the induction of fusion rate among DC/tumor cells (^i^p>0.05). Notably, PEG induced a higher fusion rate among lymphocyte/tumor cells compared with electroporation (^j^p<0.001). BSB, biotin-streptavidin-biotin; PEG, polyethylene-glycol; DC, dendritic cell. Data are presented as the mean ± standard deviation.
